# Rising to the Challenge: An ID Provider–Led Initiative to Address Penicillin Allergy Labels at a Large Veterans Affairs Medical Center

**DOI:** 10.1093/ofid/ofae396

**Published:** 2024-07-11

**Authors:** Reuben J Arasaratnam, Jessica M Guastadisegni, Marcus A Kouma, Daniel Maxwell, Linda Yang, Donald F Storey

**Affiliations:** Veterans Affairs North Texas Health Care System, University of Texas Southwestern Medical Center, Dallas, Texas, USA; Pharmacy Department, Veterans Affairs North Texas Health Care System, Dallas, Texas, USA; Pharmacy Department, Veterans Affairs North Texas Health Care System, Dallas, Texas, USA; Veterans Affairs North Texas Health Care System, University of Texas Southwestern Medical Center, Dallas, Texas, USA; Pharmacy Department, Veterans Affairs North Texas Health Care System, Dallas, Texas, USA; Veterans Affairs North Texas Health Care System, University of Texas Southwestern Medical Center, Dallas, Texas, USA

**Keywords:** amoxicillin challenge, beta-lactam, delabeling, penicillin allergy, veterans affairs

## Abstract

**Background:**

Given the negative consequences associated with a penicillin allergy label, broader penicillin allergy delabeling initiatives are highly desirable but hindered by the shortage of allergists in the United States. To address this problem at our facility, the infectious diseases section introduced a quality improvement initiative to evaluate and remove allergy labels among inpatient veterans.

**Methods:**

Between 15 November 2022 and 15 December 2023, we identified inpatients with a penicillin allergy label. We subsequently interviewed eligible candidates to stratify penicillin allergy risk and attempt to remove the allergy label directly via chart review, following inpatient oral amoxicillin challenge or outpatient community care allergy referral. Delabeling outcomes, subsequent penicillin-class prescriptions, and relabeling were tracked after successful allergy label removal.

**Results:**

We screened 272 veterans, of whom 154 were interviewed for this intervention. A total of 53 patients were delabeled: 26 directly, 23 following oral amoxicillin challenge, and 4 following outpatient allergy referrals. Of the patients who were delabeled, 25 received subsequent penicillin-class prescriptions. No adverse reactions occurred following inpatient oral amoxicillin challenges. Patients with a low-risk penicillin allergy history were more likely to undergo a challenge if admitted with an infectious diseases–related condition. Only 1 inappropriate relabeling event occurred during the study period, which was subsequently corrected.

**Conclusions:**

An infectious diseases provider–led initiative resulted in penicillin allergy label removal in more than one third of inpatients evaluated using direct removal or oral amoxicillin challenge. Efforts focused on patients who had been admitted for infections were particularly successful.

## Teaser

An inpatient, infectious diseases provider–led initiative successfully removed more than one third of penicillin allergy labels from those evaluated. Efforts focused on patients who had been admitted for infections were particularly successful.

A penicillin allergy label is the most frequent and problematic drug allergy listing in the electronic health record (EHR), with reported prevalence as high as 15% in patients hospitalized in the United States [1]. The individual- and health systems–level harms of an unverified penicillin allergy label are numerous and include development of infections such as methicillin-resistant *Staphylococcus aureus* and *Clostridioides difficile* [2], increased all-cause mortality [3], longer hospital stays, and higher costs associated with health care [4]. Because the vast majority of patients who report a penicillin allergy are subsequently shown to tolerate penicillins after formal testing [5], it is imperative to seek removal of penicillin allergy labels, or “delabel,” where possible—a recommendation endorsed by national allergy society guidance [6, 7], societies of infectious diseases and pharmacy [8], and the U.S. Centers for Disease Control and Prevention [9].

For several decades, outpatient referral to allergy specialists for penicillin skin testing with or without subsequent oral amoxicillin challenge has been the typical pathway for penicillin allergy delabeling [10]. However, the lack of allergy specialists across the United States and their presence predominantly in urban academic centers are limiting factors to this modality of penicillin allergy evaluation [11, 12]. More recently, the use of internationally validated point-of-care decision tools in conjunction with direct oral amoxicillin challenge has emerged as a safe and effective strategy for penicillin allergy label removal in select low-risk patients [13], an approach that would potentially facilitate more widespread penicillin allergy delabeling efforts by nonallergy providers across multiple health care settings.

As the largest integrated health care system in the United States, the Veterans Health Administration (VHA) has faced similar challenges in relation to penicillin allergy evaluation. A review of the published literature on penicillin allergy evaluation programs in the VHA revealed pharmacist evaluation, with support from allergy specialists, as the most frequently used modality, whereas use of direct oral amoxicillin challenge by nonallergy providers was limited [14].

The Antimicrobial Stewardship Task Force conducted a national survey of 138 Veterans Affairs (VA) facilities in 2020, where 28 (20%) reported having a formal process for evaluating penicillin allergy, with only 17 of these 28 (61%) reporting ready access to penicillin skin testing.

Our VA facility has not had onsite allergy support since 2016. In response to the growing clinical need for penicillin allergy evaluation, we implemented an infectious diseases (ID) provider–led penicillin allergy quality improvement initiative with a focus on inpatient direct oral amoxicillin challenge. Here, we report the results of this initiative.

## METHODS

### Study Setting and Approval

The Dallas VA Medical Center is a 1A critical access facility within the VA North Texas Health Care System that consists of 248 acute care inpatient beds and serves 206 000 veterans in northern Texas and southern Oklahoma. The staffing within the ID section includes 5 ID physicians, 1 ID/critical care physician, 2 advanced practice providers, and 3 ID pharmacists. An existing antimicrobial stewardship program maintains updated facility antibiograms and guidelines for common infectious syndromes; monitors antibiotic usage; and promotes appropriate prescriptions through prospective audits and feedback, formulary restriction, and provider education. Our facility does not have any access to onsite allergy/immunology providers, and formal penicillin allergy evaluation is only possible through referral for (non-VA) community care allergy evaluation (CCAE). Before this intervention, there was no formal process for evaluating or removing penicillin allergy labels from inpatients.

In June 2021, our facility's Quality, Safety, and Value (QSV) section granted approval for this project as quality improvement with nonresearch designation, waiving the need for a formal review by the institutional review board. We used the Lean Six Sigma DMAIC (Define, Measure, Analyze, Improve, Control) methodology for our process [15]. A multidisciplinary team comprising representatives from nursing, hospital medicine, QSV, pharmacy, and ID sections met approximately bimonthly for more than a year to formulate a standard operating procedure for evaluating and removing penicillin allergies for our facility. This ultimately resulted in the creation of a penicillin allergy evaluation team—Penicillin Allergy Evaluation and Response Team (PEN-ALERT)—in the fall of 2022. The PEN-ALERT consisted of 1 supervising ID physician, 2 ID pharmacists, and a grant-funded clinical coordinator (who worked between 5 and 15 hours per week for the duration of the initiative). The inpatient ID consultation team, including ID attending physicians, an ID fellow, and an advanced practice provider, provided additional support for this initiative.

### Screening, Inclusion, and Penicillin Allergy Risk Stratification

Between 15 November 2022 and 15 December 2023, we identified inpatients who were admitted for any reason to the acute medical floors with an EHR-listed penicillin allergy (defined as a documented allergy to any of the following medications: penicillin, amoxicillin, ampicillin, amoxicillin-clavulanate, ampicillin-sulbactam, or nafcillin). We identified these patients predominantly through our ID consultation service and a health informatics dashboard, which lists all inpatients with a β-lactam allergy. Because of limited time and resources, we were unable to identify all inpatients with a listed penicillin allergy. An initial chart review was performed to determine interview candidacy based on planned date of discharge, supervising ID physician availability, and presence of complex comorbidities or other factors that could have impacted the interview and ability to perform a potential amoxicillin challenge (eg, cognitive impairment).

A member of the PEN-ALERT then performed an in-depth EHR review and a face-to-face interview to investigate the patient's penicillin allergy using a locally developed questionnaire (Supplementary Figure 1). Following the interview, the patient's penicillin allergy was classified (as no increased risk, intolerance, low risk, moderate-high risk, or very high risk) and patients were offered direct removal of the label (this required history or evidence of safe receipt of a penicillin since index reaction), oral amoxicillin challenge, CCAE after discharge, or no intervention (Table 1). This risk classification criteria was developed in house based on a feasibility study we have previously published [16]. Concurrently, we calculated the PEN-FAST score [17] for patients that were interviewed. We respected patients’ wishes to decline interview regarding their penicillin allergy. We also honored patients’ preferences for CCAE following discharge regardless of risk stratification.

**Table 1.** Penicillin Allergy Risk Stratification and Recommendation Algorithm

Risk Stratification	Typical Assessment Features	Recommended Action^a^
No increased risk	Prior index reaction with proven safe receipt of a penicillin after, family history	Remove penicillin allergy label; no need for challenge. Provide delabeling card, document, and alert Patient Aligned Care Team
Intolerance	Nonallergy symptoms (eg, gastrointestinal upset)	As above or direct oral amoxicillin challenge^b^
Low risk	Self-limited rash/pruritus (at any point), urticaria only >10 y ago, unknown reaction >10 y ago	Direct oral amoxicillin challenge^b^
Moderate-high risk	Anaphylaxis or angioedema at any point Any of the following within last 10 y: urticaria, bronchospasm, loss of consciousness, severe gastrointestinal symptoms, or unknown reaction	Consider Community Care Consult for Allergy and penicillin skin testing
Very high risk	Severe cutaneous adverse reactions, delayed severe reactions (eg, AGEP, SJS, DRESS, TEN), serum sickness, acute interstitial nephritis, DILI	Do not challenge with amoxicillin; infectious diseases can be consulted for alternative antimicrobials and Community Care Consult for Allergy can be considered for further opinion

Abbreviations: AGEP, acute generalized exanthematous pustulosis; DILI, drug-induced liver injury; DRESS, drug rash with eosinophilia and systemic symptoms; SJS, Stevens-Johnson syndrome; TEN, toxic epidermal necrolysis.

^a^This risk stratification is a guide only. Providers ought to use their discretion to accommodate individual circumstances, including patient preferences for outpatient allergy evaluations.

^b^Patients that successfully underwent oral amoxicillin challenge were also provided with a delabeling card, had their allergy documentation adjusted, and notification sent to the Patient Aligned Care Team.

### Oral Amoxicillin Challenge

Eligibility criteria for oral amoxicillin challenge were previously published in a feasibility study using retrospective data from our facility [16]. These criteria are a low-risk or intolerance history, sufficient time to perform and monitor challenge, and absence of the following: inability to provide informed consent because of altered mental status/cognitive impairment, severe cardiac or respiratory failure, severe sepsis or shock, suspected drug reaction at the time of admission, new-onset rash, nausea/vomiting, abdominal pain, or inability to take oral medications. Final eligibility determination was at the discretion of the supervising ID physician. β-Blockers, use of antihistamines, and systemic steroids were not considered contraindications to proceeding with a challenge. Before proceeding with a challenge, verbal informed consent was obtained from the patient, along with acquired agreements with the patient's inpatient primary physician and nursing staff.

Baseline vital signs were obtained if not already documented 4 hours before the challenge. The oral challenge consisted of a single dose of amoxicillin (500 mg) and observation for a minimum of 30 minutes by a member of the PEN-ALERT. This direct monitoring approach by our team was chosen to allay nursing concerns iterated in our multidisciplinary QSV meetings. The patient was required to remain on the floor 1 hour following a challenge, after which they were reviewed by a supervising ID physician for evidence of any reaction. Patients were educated on allergic symptoms to monitor for, including development of rash and difficulty breathing. Our facility's rapid response team and medications to manage a hypersensitivity reaction (including epinephrine) were available at the time of the challenge. Challenges were performed only between 7 Am and 5 Pm, Monday through Friday, and a supervising ID physician was immediately available onsite throughout the process.

Patients who were successfully delabeled (either directly or via oral amoxicillin challenge) were counseled on the results of the evaluation, provided a delabeling card, and had their penicillin allergy removed from the EHR. Following the challenge, we performed a prospective chart review 48 hours later to look for evidence of any delayed cutaneous reaction. In the event of an acute or delayed reaction following the challenge, the allergy listing was to be amended, and the patient was offered CCAE.

### Patient Consent Statement

Formal written consent was not required because this project was designated quality improvement. Verbal informed consent was obtained before any interviews or delabeling.

### Referral for CCAE

For veterans requiring or requesting CCAE, the supervising ID physician placed the referral through our community care office, and the PEN-ALERT tracked the consult outcomes. Before placing the order, we checked that veterans were agreeable to this referral, counseled them on the likelihood of 2 clinic visits (1 for evaluation and 1 for testing), and ensured they had sufficient means (eg, travel support) to attend appointments. Following the completion of CCAE, the PEN-ALERT reviewed outside records and amended the drug allergy module of the patient's EHR as needed.

### Data Collection and Outcomes

We collected data on patient demographics, admission diagnoses, details of index penicillin allergy reaction, comorbidities, and outcomes of the penicillin allergy assessment. The primary outcome was the number of patients with successful removal of their penicillin allergy label between 15 November 2022 and 15 December 2023. We explored the following secondary outcomes: safety and outcomes of oral amoxicillin challenges and factors predicting receipt of oral amoxicillin challenge in the low-risk group. Ninety days after the end of the intervention period (14 March 2024), we reviewed CCAE outcomes, use of penicillins (as inpatient or outpatient) after label removal, and readdition of the penicillin allergy label to the patient's list of allergies in the EHR (relabeling events). Relabeling events were defined as “appropriate” if subsequent use of a penicillin-class antibiotic led to a reaction and “inappropriate” if there was no such evidence to justify readdition of the penicillin allergy label.

Data were summarized using descriptive statistics. To compare differences in the characteristics between veterans in the low-risk group who received oral amoxicillin challenge and those who did not, we used χ^2^ and Fisher exact tests (for categorical variables) and Mann-Whitney *U* test (for continuous variables). Statistical significance was defined as *P* < .05.

## RESULTS

**Figure 1.**
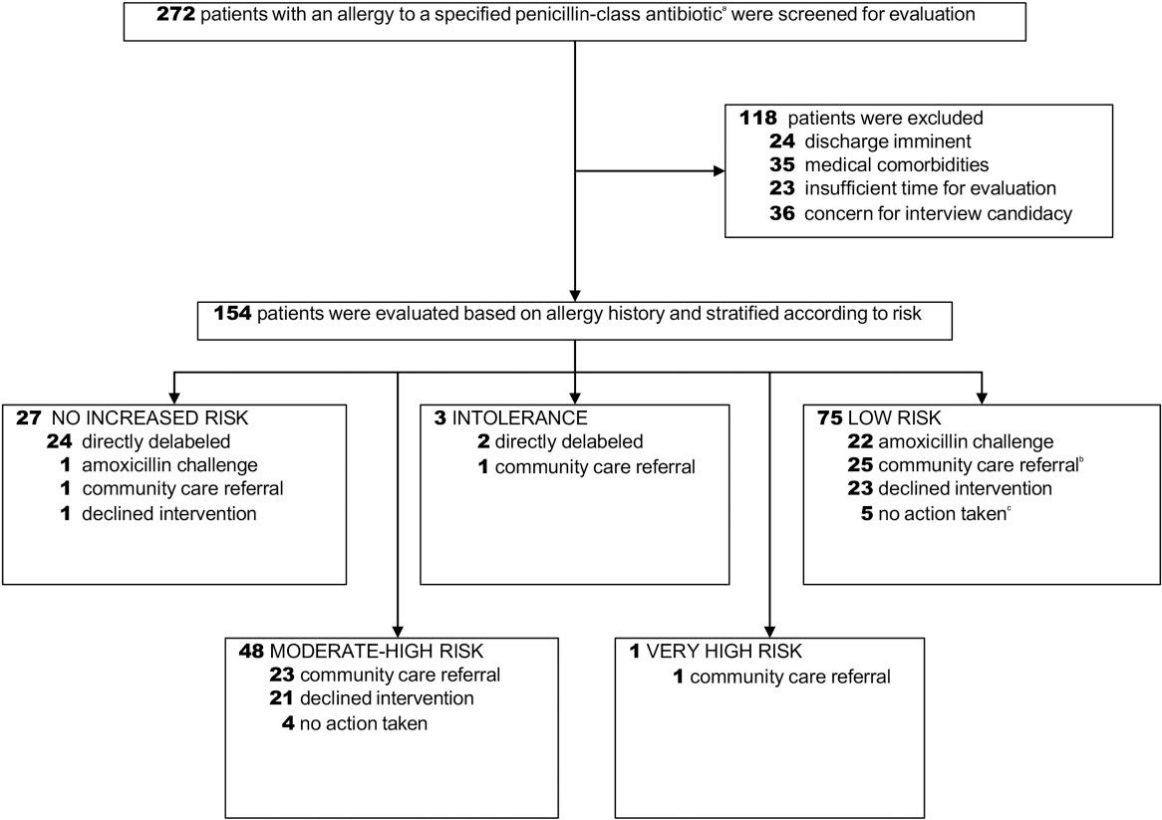
Flowchart of patients with listed penicillin allergy who were screened, interviewed, and delabeled in the study. ^a^These included penicillin, amoxicillin, ampicillin, nafcillin, ampicillin-sulbactam or amoxicillin/clavulanic acid. ^b^Low-risk patients may have been referred to the community because of 1 or more of the following: patient-preferred formal outpatient assessment, physician concerns about acute medical comorbidities, or insufficient staffing/time. ^c^No action taken because of 1 or more of the following: patient expired; social, geographic, or medical factors limiting patient's ability to access community care.

During the study period, there were 582 unique patients admitted with a penicillin allergy label to the acute medicine floors of our facility. Our team screened a total of 272 inpatients with a listed penicillin allergy (Figure 1). Of those, 118 were excluded by the initial screen, resulting in 154 patients who were evaluated in person. Based on our facility's QSV Lean Six Sigma Process, we had a minimum target of performing 10 oral amoxicillin challenges to meet the requirements of the Improve phase of the DMAIC process (completed in February 2023), after which our facility granted us approval to transition into the Control phase of the DMAIC process.

### Characteristics of Inpatient Veterans With a Penicillin Allergy who Were Interviewed

In Table 2, we show the baseline demographic characteristics of the 154 inpatient veterans with a penicillin allergy that our team interviewed. Most patients were male (90.9%) and White (60.4%), with an average age of 67 years. The median Charlson Comorbidity Index was 4 (interquartile range [IQR] 3–6). This was driven primarily by a high prevalence of diabetes mellitus (45.4%), congestive heart failure (25.3%), solid tumors (20.8%), and chronic obstructive pulmonary disease (20.1%). Half of the patients our team evaluated were admitted for infection-related reasons or received antibiotic therapy during their admission. Of the penicillin-class antibiotics listed as an allergy, penicillin was the most frequently listed (91.6%), followed by amoxicillin (7.8%) and amoxicillin-clavulanate (0.6%). The most frequent patient-reported reaction was cutaneous reaction (50.6%), followed by swelling (24.7%) and shortness of breath (18.8%). Only 3 patients (1.9%) reported anaphylaxis, and no patients directly reported a diagnosis of severe cutaneous adverse reaction. Of all reactions, 16% were classified as unknown. For >90% of those interviewed, the index date for a reaction to penicillin was >10 years ago. Using our risk stratification algorithm, we classified these 154 patients as follows: no increased risk (n = 27), intolerance (n = 3), low risk (n = 75), moderate-high risk (n = 48), or very high risk (n = 1).

**Table 2.** Characteristics of Patients Interviewed With a Penicillin Allergy

Characteristic	N = 154
Age, median (IQR), y	67 (60–73)
Sex, No. (%)	
Female	14 (9.1%)
Male	140 (90.9%)
Race and ethnicity, No. (%)	
White	93 (60.4%)
Black or African American	46 (29.9%)
Other	15 (9.7%)
Charlson Comorbidity Index score, median (IQR)	4 (3–6)
Charlson Comorbidity Index components, no. (%)^a^	
Myocardial infarction	12 (7.8%)
Congestive heart failure	39 (25.3%)
Peripheral vascular disease	19 (12.3%)
Cerebrovascular accident or transient ischemic attack	14 (9.1%)
Hemiplegia	3 (1.9%)
Chronic obstructive pulmonary disease	31 (20.1%)
Diabetes without complications	49 (31.8%)
Diabetes with end organ damage	21 (13.6%)
Moderate or severe renal disease	5 (3.2%)
Mild liver disease	6 (3.9%)
Moderate or severe liver disease	3 (1.9%)
Peptic ulcer disease	6 (3.9%)
Localized solid tumor	28 (18.2%)
Metastatic solid tumor	4 (2.6%)
Leukemia	0 (0.0%)
Lymphoma	2 (1.3%)
Dementia	1 (0.6%)
Rheumatic or connective tissue disease	8 (5.2%)
HIV or AIDS	2 (1.3%)
Reason for admission, no. (%)	
Infection related or treated with antibiotics	77 (50.0%)
Noninfection related, no antibiotics received	77 (50.0%)
Reported allergy label, no. (%)	
Penicillin	141 (91.6%)
Amoxicillin	12 (7.8%)
Amoxicillin-clavulanate	1 (0.6%)
Ampicillin	0 (0%)
Ampicillin-sulbactam	0 (0%)
Nafcillin	0 (0%)
Health professional who entered allergy, no. (%)	
Medical doctor	34 (22.1%)
Physician assistant/nurse practitioner	26 (16.9%)
Pharmacist	26 (16.9%)
Nurse	46 (29.9%)
Other	22 (14.3%)
Observed allergic reaction, no. (%)	4 (2.6%)
Patient-reported reaction, no. (%)^a^	
Unknown	25 (16.2%)
Cutaneous reaction^b^	78 (50.6%)
Swelling	38 (24.7%)
Shortness of breath	29 (18.8%)
Other	21 (13.6%)
Gastrointestinal side effects	6 (3.9%)
Anaphylaxis	3 (1.9%)
Treatment given for reaction, no. (%)	
Yes	53 (34.4%)
No	20 (13.0%)
Unknown	81 (52.6%)
Timing since index reaction, no. (%)	
Unknown	4 (2.6%)
<5 y	5 (3.2%)
5–10 y	2 (1.3%)
>10 y	143 (92.9%)
Concurrent antibiotic allergies listed, median (IQR)	0 (0–0)
Concurrent nonantibiotic allergies listed, median (IQR)	1 (0–2)
Risk stratification, no. (%)^c^	
No increased risk	27 (17.5%)
Intolerance history	3 (1.9%)
Low risk	75 (48.7%)
Moderate-high risk	48 (31.2%)
Very high risk	1 (0.6%)
PEN-FAST^d^ score	
0 points	16 (10.4%)
1–2 points	85 (55.2%)
3 points	52 (33.8%)
4–5 points	1 (0.6%)

Abbreviations: IQR, interquartile range.

^a^Categories are not mutually exclusive because patients may have had more than 1 comorbidity or symptom listed; percentages may add to more than 100%.

^b^We included acute generalized exanthematous pustulosis, drug rash with eosinophilia and systemic symptoms, Stevens-Johnson syndrome, and toxic epidermal necrolysis in this category; however, no patients verbally reported this diagnosis in our cohort.

^c^Patients with confirmed safe receipt of any penicillin-class antibiotic other than piperacillin/tazobactam after the index date were classified as “no increased risk.” Those who received piperacillin-tazobactam were reclassified as “no increased risk” only if the original history was consistent with a low-risk allergy.

^d^The PEN-FAST score is a penicillin allergy clinical decision rule, the points being as follows: PEN, penicillin allergy reported by patient; F, 5 y or less since reaction (2 points); A, anaphylaxis or angioedema (2 points); S, severe cutaneous adverse reaction (2 points); T, treatment required for reaction (1 point). 0 points: very low risk of positive penicillin allergy test (<1%); 1–2 points: low risk (5%); 3 points: moderate risk (20%); 4–5 points: high risk (50%).

The calculated PEN-FAST score for these patients is included in Table 2, where almost two thirds of patients scored 2 points or less, which is indicative of low risk.

### Penicillin Allergy Delabeling

**Figure 2.**
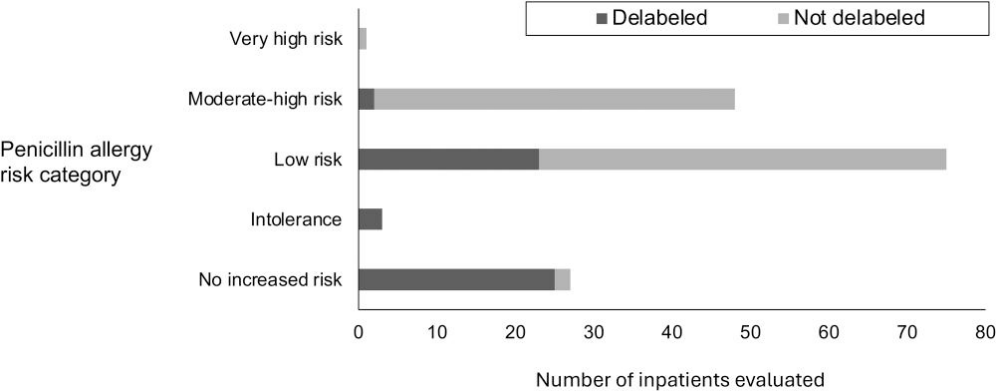
Success of penicillin allergy removal, stratified by risk category.

A total of 53 patients had their penicillin allergy removed over the course of the study (direct delabel, n = 26; amoxicillin challenge, n = 23; CCAE, n = 4). When stratified by penicillin allergy risk category, the proportion of patients delabeled following interview was highest in the no-increased-risk and intolerance groups, followed by the low-risk and then moderate-high-risk groups (Figure 2). During the follow-up period, our team noted only 1 of the 53 patients had an inappropriate relabeling event (which was corrected). Twenty-five patients who were delabeled subsequently received at least 1 prescription for penicillin-based therapy (ampicillin, ampicillin-sulbactam, piperacillin-tazobactam, amoxicillin, amoxicillin/clavulanate) at a median of 1 day (IQR 1–4) after label removal.

### Safety and Outcomes of Oral Amoxicillin Challenges

Twenty-three oral amoxicillin challenges were performed: 22 in the low-risk group and 1 in the no-increased-risk group (per patient request). No nursing or primary provider resisted any of the proposed oral amoxicillin challenges. No reactions were observed in any of these patients based on our monitoring protocol (including no delayed reactions), and all patients had their penicillin allergy labels removed. In no instance did the amoxicillin challenge interfere with planned patient care processes (eg, diagnostic imaging) or length of stay. The median PEN-FAST score of patients who underwent a challenge was 1 (IQR 1–1).

Next, we explored baseline differences in the patient characteristics of those in the low-risk group (n = 75) who either proceeded with the challenge (n = 22) or did not (n = 53). There were no significant differences between the groups in terms of age, sex, race and ethnicity, or Charlson Comorbidity Index (Supplementary Table 1). However, patients who received an oral amoxicillin challenge compared with those who did not were more likely to have been admitted for an infection-related reason that was treated with antibiotics (86.4% vs 39.6%, *P* < .01).

### Comparison of Facility-derived Penicillin Allergy Risk Classification to PEN-FAST Score

The median PEN-FAST scores, stratified by our penicillin allergy risk classification algorithm, are shown in Supplementary Table 2. In general, there was alignment between our risk classification and the PEN-FAST score with our low-risk patients having a median PEN-FAST score of 1 (IQR 1–1) and moderate-high risk patients having a median PEN-FAST score of 3 (IQR 3–3).

### Outcomes of CCAE

Fifty-one veterans were referred for CCAE. At the time of final analysis, 26 patients had been successfully scheduled, 11 had completed an initial consultation, and 4 had had their penicillin allergy label successfully removed. These 4 patients were in the following risk categories: intolerance (n = 1), low risk (n = 1), and moderate-high risk (n = 2). The majority of incomplete CCAEs were due to an inability to contact veterans for scheduling, veterans subsequently declining services before scheduling, and veterans failing to present for scheduled appointments.

## DISCUSSION

In this quality improvement initiative, we successfully introduced an inpatient penicillin allergy delabeling program at a large VA facility that does not have onsite allergy physician support. More than 150 veterans were interviewed in person, and more than one third of these had their penicillin allergy label successfully removed during an inpatient stay, predominantly via direct delabeling or following oral amoxicillin challenge. Delabeling was followed by subsequent use of penicillin-based therapy in nearly half of the patients delabeled, which highlights the rapid actionability of this intervention. We also calculated PEN-FAST scores on all veterans evaluated and are the first to report these data in the U.S. veteran population. In general, there was alignment between our risk classification system and PEN-FAST score, and all patients who underwent direct oral amoxicillin challenges had a PEN-FAST score of 1 or less.

To date, very few published reports within the VHA exist regarding penicillin allergy removal initiatives that incorporate the use of oral amoxicillin challenge [14]. Caturano and colleagues [18] chronicled a series whereby 22 of 136 patients admitted to the Miami VA Medical Center were delabeled using this approach. Nguyen and colleagues [19] implemented an initiative in 128 veterans seen in the emergency department at the VA Greater Los Angeles Healthcare System in which they were able to remove penicillin allergy labels in 40 patients via direct delabeling and in 16 patients via amoxicillin challenge. In contrast to our situation, both facilities had onsite allergy specialist support and were able to offer in-house referrals to an allergy clinic for penicillin skin testing. A planned VHA expansion of penicillin allergy evaluation services led by pharmacists, independent of onsite allergy support, has recently been launched [20]. However, uptake by the VA facility is voluntary and contingent on pharmacy and physician champion support, which may be a limiting factor for implementation.

Specific contextual challenges exist in the inpatient setting when performing oral amoxicillin challenges, particularly in an elderly population with complex and numerous comorbidities. In another VA facility study, Alagoz et al. [21] found that fear of inducing hypersensitivity reaction, lack of ownership of penicillin allergy evaluation process, and competing patient care priorities were major obstacles to delabeling. In our current study, fewer than one third of eligible low-risk patients (22 of 75) underwent challenges. Lack of provider availability for monitoring, patients declining or deferring evaluation, and complexity of medical comorbidities were the most frequent reasons. Consistent with prior literature [22], amoxicillin challenges occurred more frequently in patients who were admitted for an infectious disease condition compared with a noninfectious disease condition. This may reflect a higher motivation to address penicillin allergy status from our veterans when the immediate benefits were evident. To maximize the impact of our efforts, we have now focused resources on performing oral amoxicillin challenges predominantly in patients admitted with active infectious disease conditions that require antibiotic therapy.

An unexpected finding in our study was the complexity of the veteran CCAE process. Non-VA community care providers are essential, especially when there are long travel times or when a needed service is not available, as is the case at many other VA facilities with respect to allergy/immunology. The Maintaining Internal Systems and Strengthening Integrated Outside Networks Act of 2018 was designed to improve access and timeliness of care to community providers, and it is estimated that 2.6 million veterans currently access care through this process [23]. However, there are several steps to coordinating care with outside non-VA providers [24], including tracking referral status and accessing records. This is particularly pertinent for penicillin allergy evaluation, where records of penicillin allergy removal must be translated to an update of the drug allergy module in the EHR to confer benefit to the patient. To date, we are not aware of any published reports of CCAE for penicillin allergy evaluation within the VHA, which may be a future area of study.

Considering this is a new process at our facility, our approach to penicillin allergy risk classification and delabeling was conservative and guided by local factors, such as an older patient population with multiple comorbidities, absence of onsite allergy support, and limited monitoring capacity. Further limitations in our study include the use of oral amoxicillin challenges among veterans only in the lowest risk groups, (ie, those with a median PEN-FAST score of 1) and the lack of access to penicillin skin testing. These factors limit the ability to assess how accurately our classification system identifies those patients in moderate-high or very high-risk groups (ie, PEN-FAST scores >2), and our findings should not be extrapolated outside the lower risk groups studied here. Staffing limitations prevented us from evaluating all potential patients admitted with a penicillin allergy during the intervention period. Because no positive reactions occurred following amoxicillin challenges, we are unable to report outcomes pertaining to this. Although only 1 inappropriate relabeling event occurred, our follow-up period was limited to 3 months after the intervention. Longer term follow-up (beyond a year) is warranted to assess the sustained benefit of this initiative.

In summary, we demonstrate the successful implementation of an ID provider–led inpatient quality improvement initiative to evaluate and remove penicillin allergies in a U.S. veteran population with complex comorbidities. We also observed an immediate clinical benefit to delabeling with respect to subsequent antibiotic selection. These findings add to the body of literature supporting the inpatient use of oral amoxicillin challenges by nonallergy providers as a means of expanding access to penicillin allergy evaluation.

## Supplementary Material

ofae396_Supplementary_Data

## Supplementary Data

Supplementary materials are available at *Open Forum Infectious Diseases* online. Consisting of data provided by the authors to benefit the reader, the posted materials are not copyedited and are the sole responsibility of the authors, so questions or comments should be addressed to the corresponding author.

## Notes

***Acknowledgments***. We thank the Quality, Safety, and Value Section at the VA North Texas Health Care System for their support in this quality improvement initiative. We thank Maximillian Masuda, Jazmin Criss, and Sindi Sanchez at the Dallas VA Research Corporation for their assistance in interviewing patients and data collection. We thank the University of Texas Southwestern Medical Center Division of Allergy and Immunology for their initial review and feedback on our protocols. We thank Allison Herrera for her thoughtful input and comments regarding this manuscript.

***Author contributions***. The authors confirm their contribution to the paper as follows: study conception and design: R. J. A., J. M. G., M. A. K.; administration and implementation: R. J. A., J. M. G., M. A. K., D. F. S., D. M., L. Y.; data collection: R. J. A., J. M. G., M. A. K.; draft manuscript preparation: R. J. A., J. M. G., M. A. K., D. M., L. Y., D. F .S. All authors reviewed the results and approved the final version of the manuscript. Overall work was supervised by R. J. A.

***Financial support*.** This work was supported by the National Academy of Medicine of the National Academy of Sciences under award number 2000001346.

## References

[1] Lee CE, Zembower TR, Fotis MA, et al The incidence of antimicrobial allergies in hospitalized patients: implications regarding prescribing patterns and emerging bacterial resistance. Arch Intern Med 2000; 160:2819–22. 11025792 10.1001/archinte.160.18.2819

[2] Blumenthal KG, Lu N, Zhang Y, Li Y, Walensky RP, Choi HK. Risk of methicillin resistant Staphylococcus aureus and Clostridium difficile in patients with a documented penicillin allergy: population based matched cohort study. BMJ 2018; 361:k2400. 29950489 10.1136/bmj.k2400 PMC6019853

[3] Blumenthal KG, Lu N, Zhang Y, Walensky RP, Choi HK. Recorded penicillin allergy and risk of mortality: a population-based matched cohort study. J Gen Intern Med 2019; 34:1685–7. 31011962 10.1007/s11606-019-04991-y PMC6712108

[4] Mattingly TJ II, Fulton A, Lumish RA, et al The cost of self-reported penicillin allergy: a systematic review. J Allergy Clin Immunol Pract 2018; 6:1649–1654.e4. 29355644 10.1016/j.jaip.2017.12.033

[5] Sacco KA, Bates A, Brigham TJ, Imam JS, Burton MC. Clinical outcomes following inpatient penicillin allergy testing: a systematic review and meta-analysis. Allergy 2017; 72:1288–96. 28370003 10.1111/all.13168

[6] Khan DA, Banerji A, Blumenthal KG, et al Drug allergy: a 2022 practice parameter update. J Allergy Clin Immunol 2022; 150:P1333–1393. 10.1016/j.jaci.2022.08.028 36122788

[7] American Academy of Allergy, Asthma, and Immunology Penicillin allergy evaluation should be performed proactively in patients with a penicillin allergy label—a position statement of the American Academy of Allergy, Asthma & Immunology. 31 August 2023. Available at: https://education.aaaai.org/sites/default/files/media/2023-09/Penicillin-Allergy-Position-Statement_Approved-08-31-2023.pdf. Accessed 8 October 2023 10.1016/j.jaip.2023.09.045 37838278

[8] Barlam TF, Cosgrove SE, Abbo LM, et al Implementing an antibiotic stewardship program: guidelines by the Infectious Diseases Society of America and the Society for Healthcare Epidemiology of America. Clin Infect Dis 2016; 62:e51–77. 27080992 10.1093/cid/ciw118 PMC5006285

[9] Centers for Disease Control and Prevention . Is it really a penicillin allergy? Available at: https://www.cdc.gov/antibiotic-use/community/pdfs/penicillin-factsheet.pdf. Accessed 2 October 2023.

[10] Macy E, Adkinson NF Jr. The evolution of our understanding of penicillin allergy: 1942–2022. J Allergy Clin Immunol Pract 2023; 11:405–13. 36116763 10.1016/j.jaip.2022.09.006

[11] Arasaratnam RJ, Chow TG, Liu AY, Khan DA, Blumenthal KG, Wurcel AG. Penicillin allergy evaluation and health equity: a call to action. J Allergy Clin Immunol Pract 2023; 11:422–8. 36521831 10.1016/j.jaip.2022.12.001

[12] Mancini CM, Fu X, Zhang Y, et al Penicillin allergy evaluation access: a national survey. Clin Infect Dis 2020; 71:2972–5. 32421192 10.1093/cid/ciaa567 PMC7947974

[13] Copaescu AM, Vogrin S, James F, et al Efficacy of a clinical decision rule to enable direct oral challenge in patients with low-risk penicillin allergy: the PALACE randomized clinical trial. JAMA Intern Med 2023; 183:944–52. 37459086 10.1001/jamainternmed.2023.2986 PMC10352926

[14] Kouma MA, Guastadisegni JM, Yang L, Maxwell DN, Storey DF, Arasaratnam RJ. Challenges and opportunities related to penicillin allergy in the Veterans Health Administration: a narrative review. Antimicrob Steward Healthc Epidemiol 2023; 3:e174. 38028897 10.1017/ash.2023.448 PMC10644167

[15] Harolds JA . Quality and safety in healthcare, part xciv: Six Sigma and Lean Six Sigma in health care. Clin Nucl Med 2023; 48:e556–8. 35044964 10.1097/RLU.0000000000004059

[16] Guastadisegni JM, Kolala MK, Criss JM, Kouma MA, Storey DF, Arasaratnam RJ. Oral amoxicillin challenges for low-risk penicillin-allergic patients at a large Veterans Affairs facility: a retrospective feasibility analysis. Antimicrob Steward Healthc Epidemiol 2024; 4:e6. 38234419 10.1017/ash.2023.532 PMC10789979

[17] Trubiano JA, Vogrin S, Chua KYL, et al Development and validation of a penicillin allergy clinical decision rule. JAMA Intern Med 2020; 180:745–52. 32176248 10.1001/jamainternmed.2020.0403 PMC7076536

[18] Caturano B, Bjork L, Temino V. Clinical pharmacist-lead amoxicillin oral challenges in low-risk penicillin allergy patients. Open Forum Infect Dis 2023; 10(suppl 2):1177.

[19] Nguyen PK, Pham MT, Calub F, Yusin J, Graber C. Penicillin de-labeling in the emergency department. Veterans Health Administration webinar. 20 May 2022. Available via VHA intranet: Available at: https://dvagov.sharepoint.com/sites/VHAPBM/ASTF/SitePages/ASP_Education.aspx. Accessed 12 December 2023

[20] Allergy to beta lactam evaluation (ABLE) . VA Diffusion Marketplace. Available at: https://marketplace.va.gov/innovations/beta-lactam-allergy-assessment-saving-lives-one-assessment-at-a-time. Accessed 8 October 2023

[21] Alagoz E, Saucke M, Balasubramanian P, Lata P, Liebenstein T, Kakumanu S. Barriers to penicillin allergy de-labeling in the inpatient and outpatient settings: a qualitative study. Allergy Asthma Clin Immunol 2023; 19:88. 37821953 10.1186/s13223-023-00842-y PMC10568923

[22] Trubiano JA, Vogrin S, Mitri E, et al The who, what, when, and where of inpatient direct oral penicillin challenge-implications for health services implementation. Clin Infect Dis 2023; 77:19–22. 36929823 10.1093/cid/ciad156

[23] Mattocks KM, Kroll-Desrosiers A, Kinney R, Elwy AR, Cunningham KJ, Mengeling MA. Understanding VA's use of and relationships with community care providers under the MISSION Act. Med Care 2021; 59(6 suppl 3):S252–8. 33976074 10.1097/MLR.0000000000001545 PMC8132889

[24] Nevedal AL, Wagner TH, Ellerbe LS, Asch SM, Koenig CJ. A qualitative study of primary care providers’ experiences with the Veterans Choice program. J Gen Intern Med 2019; 34:598–603. 30684200 10.1007/s11606-018-4810-2 PMC6445927

