# Supplementary material for: Rising to the Challenge: An ID Provider–Led Initiative to Address Penicillin Allergy Labels at a Large Veterans Affairs Medical Center
Source: Open Forum Infect Dis. 2024 Jul 11;11(8):ofae396. doi: 10.1093/ofid/ofae396 (PMC11310584; doi:10.1093/ofid/ofae396)
Supplement: ofae396_Supplementary_Data [file ofae396_supplementary_data.zip › Supplementary Table 1 and 2.docx]

### Supplementary Table 1. Comparison of patients with low-risk penicillin allergy who did and did not receive an oral amoxicillin challenge

|  | **Challenged (n = 22)** | **Not Challenged (n = 53)** | ***P* value** |
| --- | --- | --- | --- |
| **Age, median (IQR), y** | 70 (63-73)) | 69 (58-75) | .97 |
| **Male, No. (%)** | 19 (86.4%) | 48 (90.6%) | .69 |
| **White, No. (%)** | 16 (72.7%) | 35 (66.0%) | .79 |
| **Charlson Comorbidity Index score, median (IQR)** | 3 (2-3) | 2 (1-3) | .37 |
| **Reason for admission, No. (%)** | | | |
| **Infection-related or treated with antibiotics** | 19 (86.4%) | 21 (39.6%) | <.01 |
| **Noninfection-related, no antibiotics received** | 3 (13.6%) | 32 (60.4%) |  |
| **Patient-reported reaction, No. (%)^a^** | | | |
| **Unknown** | 6 (27.3%) | 12 (22.6%) | .77 |
| **Cutaneous reaction** | 11 (50.0%) | 31 (58.5%) | .61 |
| **Swelling** | 0 (0.0%) | 7 (13.2%) | .10 |
| **Shortness of breath** | 1 (4.5%) | 1 (1.9%) | .50 |
| **Other** | 4 (18.2%) | 7 (13.2%) | .72 |
| **Gastrointestinal side effects** | 0 (0.0%) | 3 (5.7%) | .55 |

^a^ Categories are not mutually exclusive as patients may have had more than one symptom listed; percentages may add to more than 100%.

**Supplementary Table 2. Comparison of facility-derived risk classification score versus PEN-FAST**

| **Penicillin allergy risk category** | **Numbers of patients** | **PEN-FAST Score, median (IQR)** |
| --- | --- | --- |
| No increased risk | 27 | 1 (1–1) |
| Intolerance | 3 | 0^a^ |
| Low risk | 75 | 1 (1–1) |
| Moderate-high risk | 48 | 3 (3–3) |
| Very high risk | 1 | 3^a^ |

^a^ No interquartile range was calculated given small sample size.
