# Supplementary figures and images for: Rising to the Challenge: An ID Provider–Led Initiative to Address Penicillin Allergy Labels at a Large Veterans Affairs Medical Center
Source: Open Forum Infect Dis. 2024 Jul 11;11(8):ofae396. doi: 10.1093/ofid/ofae396 (PMC11310584; doi:10.1093/ofid/ofae396)

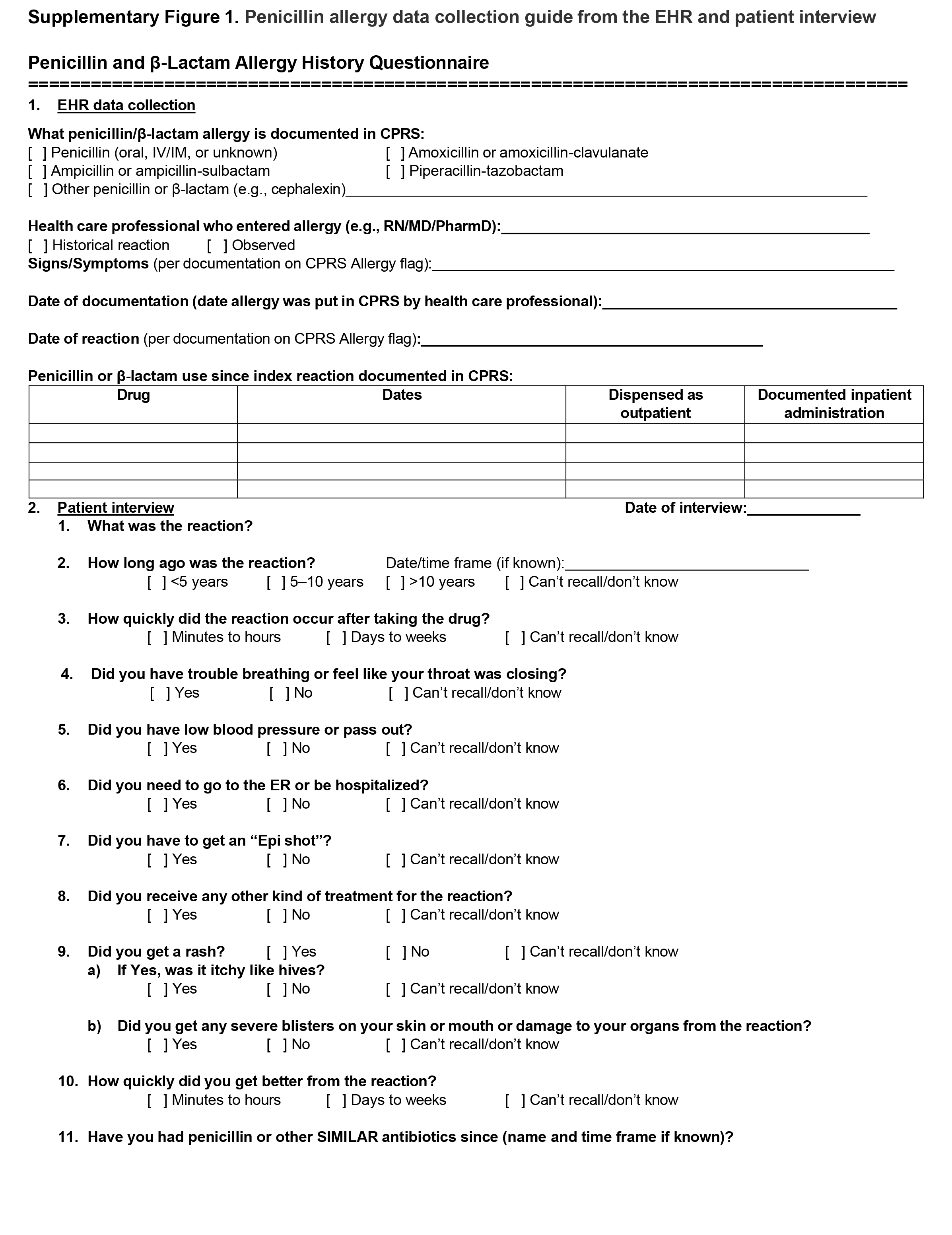

Supplement: ofae396_Supplementary_Data [file ofae396_supplementary_data.zip › Supplementary Figure 1.tif]
